# Uncomplicated Urinary Tract Infections: From an Invisible Impact to a Visible Change in Complex Care

**DOI:** 10.1093/cid/ciaf705

**Published:** 2026-02-25

**Authors:** Rafael Cantón, Ai-Nee Lim, Vanessa Cortés, Jazmín Díaz-Regañón

**Affiliations:** Servicio de Microbiología, Hospital Universitario Ramón y Cajal and Instituto Ramón y Cajal de Investigación Sanitaria (IRYCIS), Madrid, Spain; CIBER de Enfermedades Infecciosas (CIBERINFEC), Instituto de Salud Carlos III, Madrid, Spain; Department of Microbiology, Whittington Health NHS Trust, London, United Kingdom; GSK, Bogota, Colombia; GSK, Madrid, Spain

**Keywords:** uncomplicated UTI, antimicrobial resistance, antibiotics, epidemiology, treatment guidelines

## Abstract

Uncomplicated urinary tract infections can cause significant physical and emotional burden for patients, with high rates of recurrence. The challenges of treating uncomplicated urinary tract infections are exacerbated by rising antimicrobial resistance in key uropathogens, primarily *Escherichia coli*, on a global scale. Clinicians should consider drug, pathogen, and host-related factors when selecting the most appropriate therapy.

## Teaser

Uncomplicated UTIs may have high recurrence rates in subsets of patients, causing a substantial burden. Increasing antimicrobial resistance in uropathogens presents challenges for clinicians in choosing the optimal antimicrobial therapy, highlighting the need to consider drug-, pathogen-, and host-specific factors.

Uncomplicated urinary tract infections (uUTIs) have a significant impact on the quality of life of patients, with both physical and emotional well-being affected [1]. With approximately 40%–60% of adult women experiencing ≥1 urinary tract infection (UTI) during their lifetime (of whom 30%–44% experience a recurrent infection) and resistance to ≥2 classes of antibiotics seen in 15.7% of *Escherichia coli (E. coli)* urine isolates, it is necessary to understand the factors that have the greatest impact on patient experience [2–6]. Furthermore, the lack of a consensus-based reference standard to classify UTI makes treatment more complex [7]. The 2025 European Association of Urology (EAU) guidelines reclassify UTI as either “localized” or “systemic” infections, instead of uncomplicated or complicated as in previous guidance, with the updated classifications also considering patient-specific factors, such as risk of resistance, risk of allergic reaction, contraindications, or drug–drug interactions [8]. Moreover, the 2025 clinical guideline released by the Infectious Diseases Society of America (IDSA) now defines uUTI as “infection confined to the bladder in afebrile women or men” [9].

Here, we discuss the challenges and opportunities across the uUTI treatment pathway, including the importance of choosing the optimal antibiotic, the role of the evolving epidemiology, and the impact on the patient's quality of life and healthcare experience.

## Pathogenesis and Epidemiology of Urinary Tract Infections

The lifetime incidence of uUTI ranges from 37%–60% in females and 12%–20% in males, depending on many factors, including age and geographic region (ie USA vs Europe) [5, 6, 10, 11]. The elevated incidence of UTI amongst females is often attributed to the closer proximity of the urethral opening to the anus, along with a shorter urethra, allowing easier ascent of bacteria into the bladder [12]. However, this does not explain the higher prevalence of UTI in male infants under 3 months of age (20.1% amongst uncircumcised male infants vs 7.5% amongst female infants) and the similar incidence of UTI amongst male and female patients above the age of 65 years (11% vs 14%, respectively) [13, 14]. Potential explanations for this disparity include: sex-based variations in immunity and disease susceptibility; hormone levels; and changes in the physiology, structure, and function of the genitourinary system over the course of a lifetime [12].

In adult female patients experiencing UTI, >30% suffer from a recurrent episode following resolution of initial symptoms [3]. Risk factors for recurrent UTI include older age, frequent sexual intercourse, new or multiple sexual partners, the use of barrier contraceptives, spermicide use, and a history of sexually transmitted infections [3]. In adult men, recurrent UTIs usually occur in those with underlying causes, such as functional or anatomical abnormalities of the genitourinary tract [15].

Current concepts in the pathogenesis of UTI include the following: (1) *the rectal-perineal-urethral route*, where rectal microbiota colonize the perineum and urethra and ascend into the bladder [16]; (2) *the alteration of normal vaginal microbiota*, with a loss of hydrogen peroxide-producing lactobacilli resulting in persistent vaginal colonization, primarily with *E. coli* [17]; and (3) *reservoir pathogens in the epithelium of the bladder*, which can form biofilm-like intracellular bacterial communities and/or attach to the bladder epithelial cells [16].

The majority of uUTIs (∼75%) are caused by *E. coli*, with a range of other pathogens accounting for less than 10% of cases each, including *Klebsiella pneumoniae* (6%), *Staphylococcus saprophyticus* (6%), and *Proteus mirabilis* (2%) [18]. Recent clinical trial data have shown that in subgroups of patients with resistant infections (eg to nitrofurantoin), up to 90% of uUTIs are caused by *E. coli* [19, 20].

## Classification of Urinary Tract Infections

UTIs can be challenging to treat for several reasons, including the heterogeneity of the patient population, and the lack of a consensus-based reference standard to classify UTI [7, 8]. Furthermore, there is a high number of different treatment options to choose from, to which recent antibiotic approvals have been added for the first time in more than two decades [21–23].

Updated 2025 EAU and IDSA guidelines have reclassified UTI as either localized or systemic [8, 9]. The EAU guidelines define localized UTI as cystitis with typical signs or symptoms, such as frequency, urgency, and suprapubic pain, and the absence of signs or symptoms of systemic infection in either sex. Systemic UTI is defined as a UTI with signs or symptoms of systemic infections, such as fever and chills, but can also include local symptoms for pyelonephritis or prostatitis. For both types of infection, risk factors may be present in the patient and should be addressed [8]. However, the EAU guidance does not provide direction on the optimal management of uUTI for some subgroups of patients, such as patients with diabetes mellitus, older patients, or immunocompromised patients [8]; and, although IDSA 2025 reclassifies uUTI as “infection confined to the bladder in afebrile women or men, characterized by local signs and symptoms such as dysuria, frequency, urgency or suprapubic pain” [9], a review of current treatment and management options for uUTI has yet to be published, further to the guidance outlined by the IDSA for acute uUTI in 2010 [24].

For each new patient with uUTI it is important to consider the following factors: (1) *symptoms*—the clinician should identify any signs of systemic infection, tissue involvement, or even asymptomatic bacteriuria (not a uUTI) [8]; (2) *the need for culture or susceptibility testing*—traditionally, urinalysis or culture are not always carried out for simple cystitis in women. Moving forward, there could also be a role for rapid diagnostics, including antimicrobial susceptibility testing [8]; (3) *treatment considerations*—new agents have received approval for uUTI, including pivmecillinam, sulopenem, gepotidacin, and others on the horizon [21–23]. Clinicians should consider the optimal administration and duration of treatment (eg if there is any reason to treat the patient for ≥5–7 days) [8].

EAU 2025 guidelines recommend that in women with no other risk factors for systemic UTI, cystitis should be diagnosed based on a history of lower UTI symptoms, such as dysuria, frequency, and urgency, and the absence of vaginal discharge [8]. Urine dipstick testing can be used to inform a diagnosis of acute cystitis, although this is considered a weak recommendation by the guidelines. Urine cultures are strongly recommended when acute pyelonephritis is suspected, when symptoms do not resolve or recur within 4 weeks after treatment, when atypical symptoms occur in women, during pregnancy, where there are complex host factors impacting response to treatment, or if there is a concern for multi-drug resistance (MDR) [8].

## Choosing the Optimal Antimicrobial for Uncomplicated Urinary Tract Infections

Resistance to oral antimicrobials commonly used for the treatment of uUTIs, including cephalosporins and fluoroquinolones, is observed globally [25–27]. The incidence of drug-resistant phenotypes, including extended-spectrum β-lactamase (ESBL)- and carbapenemase-producing *E. coli*, are increasing annually, particularly in low- and middle-income countries where antimicrobial resistance (AMR) is exacerbated by poverty and inequality [28, 29]. Co-resistance to multiple classes of antimicrobial agents is also increasing and makes treatment more challenging [30]. Risk factors for AMR or MDR in uUTI include older age, a history of UTI and recurrent UTI, hospitalization, previous resistance, and previous antibiotic usage [31]. Moreover, the absence of rapid diagnostics for uUTI makes AMR more problematic when it comes to choosing empiric therapy. Patient risk factors must be considered, and optimal empiric choices may differ depending on location and individual circumstances (Table 1). Newer therapies may need to be considered when balancing MDR and efficient management of the patient, avoiding intravenous therapy and hospitalization wherever possible [8].

**Table 1.** Patient- and Location-specific Factors for Management of uUTIs

	Patient-specific Factors	Location-specific Factors
EAU 2025 [8]	Adverse effects/tolerability Cost Pregnancy Renal function Sex	Adverse ecological effects Availability Local antimicrobial efficacy Local antibiogram
NICE 2018 and 2024 [32, 33]	Adverse effects Age Behavioral and hygiene habits Microbiological susceptibility Patient preference Peri-menopause/menopause Pregnancy Previous antibiotic use Sex Structural alteration of the urethra Suspected cancer Recurring infection Risk of complications Symptom severity	Local antibiogram
AUA/CUA/SUFU 2022 [34]	Abnormalities of the urinary tract Bacteremia Catheter use Immunosuppression Microbiological susceptibility Peri-menopause/post-menopause Pregnancy Recurring infection	Local antibiogram
IDSA 2010^a^ and 2025 [9, 24]	Adverse effects/tolerability Age Allergies Compliance history Cost of treatment Microbiological susceptibility Patient and provider threshold for failure Pregnancy Previous antibiotic use Sex Symptom severity	Availability of drug/treatment Local practice Local antibiogram

Abbreviations: AUA, American Urological Association; CUA, Canadian Urological Association; cUTI, complicated urinary tract infection; EAU, European Association of Urology; IDSA, Infectious Diseases Society of America; NICE, National Institute for Health and Care Excellence; SUFU, Society of Urodynamics, Female Pelvic Medicine and Urogenital Reconstruction; uUTI, uncomplicated urinary tract infection.

^a^IDSA 2010 guidelines refer to acute uUTI [24]. IDSA 2025 guidelines outline treatment and management recommendations for cUTI only and therefore are not summarized here [9].

**Figure 1.**
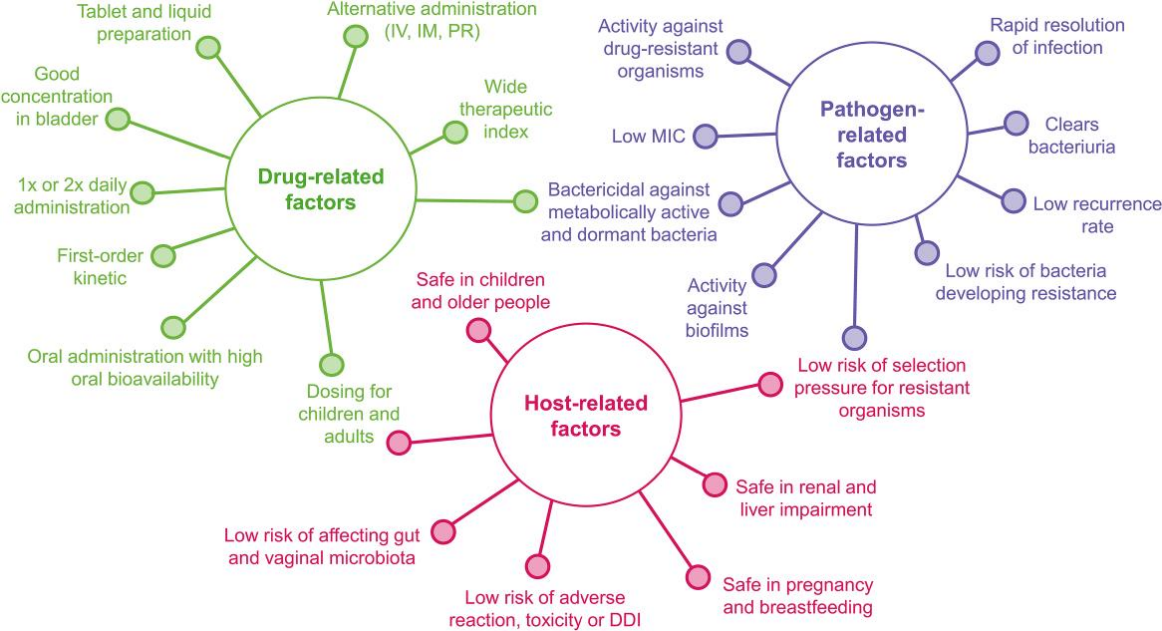
Drug-related, host-related, and pathogen-related factors for clinicians to consider when choosing the optimal antibiotic for uUTI. Abbreviations: DDI, drug–drug interaction; IM, intramuscular; IV, intravenous; MIC, minimum inhibitory concentration; PR, per rectum.

The principle for choosing an ideal antimicrobial agent for the treatment of uUTI is based on the consideration of three main categories: drug-specific, host-specific, and pathogen-specific properties (Figure 1) [35]. Drug-specific properties include good oral bioavailability, availability of both tablets and liquid preparations, availability of alternative routes of administration (eg enteral, parenteral, and rectal), low risk of drug–drug and drug–food interactions, convenient dosing administration, dosing for both children and adults, wide therapeutic index, first-order kinetic elimination, good concentration in the bladder, inexpensive to source, and a dependable supply chain. Host-specific properties include low risk of adverse reactions and toxicity, safe use in pregnancy, breastfeeding, and in those of childbearing potential, safe use in extremes of age, safe use in renal and liver impairment, and low collateral damage to patient's gut and vaginal microbiota. Pathogen-specific properties include bactericidal activity against bacteria that are metabolically active and those that are dormant, activity against drug-resistant organisms, low minimum inhibitory concentration against uropathogens, rapid resolution of infection, clears bacteriuria effectively, activity against biofilms, low recurrence rate, high genetic barrier to resistance, and low antibiotic selective pressure potential [35]. The attainment of a drug that fulfills all the listed properties is unlikely to be realized; current treatment strategies and approaches for the management of uUTI entail the optimized, judicious use of existing antimicrobial agents [8, 35].

Treatment guidelines for uUTI in Europe, the USA, and the UK are broadly similar, with some divergences in the preferred first choice antimicrobial agents and optimum treatment duration of specific antibiotics (Table 2) [8, 24 , 32–34]. Commonalities amongst acute uUTI treatment guidelines include the routine use of nitrofurantoin, fosfomycin, and pivmecillinam, limited utilization of trimethoprim or trimethoprim-sulfamethoxazole only if the risk of resistance is low, and oral cephalosporins when other recommended agents fail or are not suitable. There are stringent restrictions on the use of certain agents, such as ciprofloxacin due to the propensity for ecological collateral damage and risk of disabling and potentially long-lasting side effects, amoxicillin-clavulanate because of adverse ecological effects, and amoxicillin due to the high rates of *E. coli* resistance worldwide [8, 24 , 32]. For recurrent uUTI, routine recommended prophylaxis includes nitrofurantoin and trimethoprim. There are deviations amongst national guidelines on the use of fosfomycin, oral cephalosporins, and amoxicillin [8, 33, 34].

**Table 2.** Treatment Guidelines for Acute UTI and Recurrent UTI in Women

	EAU 2025 [8]	NICE 2018 And 2024 [32, 33]	AUA/CUA/SUFU 2022 And IDSA 2010^a^ [24, 34]
Acute uUTI in women			
First choice	Fosfomycin single dose or nitrofurantoin 5 d or pivmecillinam 3–5 d	Nitrofurantoin 3 d or trimethoprim 3 d (if low risk of resistance) If pregnant: Nitrofurantoin 7 d	Nitrofurantoin 5 d or pivmecillinam 5 d or trimethoprim 3 d (avoid if prevalence of resistance exceeds 20% or if used in previous 3 m) or fosfomycin single dose
Second choice	Cephalosporins (eg cefadroxil) 3 d If local resistance pattern for *E. coli* is <20%: Trimethoprim 5 d or co-trimoxazole 3 d	Pivmecillinam 3 d or fosfomycin single dose If pregnant: Amoxicillin 7 d or cephalexin 7 d	
Recurrent uUTI in women (prophylaxis)			
First choice	Nitrofurantoin once daily or fosfomycin once a week or trimethoprim once daily	Methenamine twice a day or trimethoprim once daily or nitrofurantoin once daily	Trimethoprim once daily or nitrofurantoin once daily or cephalexin once daily or fosfomycin every 10 d
Second choice	*If pregnant:* Cephalexin once daily or cefaclor once daily	Amoxicillin once daily or cephalexin once daily	
Duration	Usually from 3–12 m (no consensus)	Review at least every 6 m	

Abbreviations: AUA, American Urological Association; CUA, Canadian Urological Association; cUTI, complicated urinary tract infection; EAU, European Association of Urology; IDSA, Infectious Diseases Society of America; NICE, National Institute for Health and Care Excellence; SUFU, Society of Urodynamics, Female Pelvic Medicine and Urogenital Reconstruction; UTI, urinary tract infection; uUTI, uncomplicated urinary tract infection.

^a^IDSA 2010 guidelines refer to acute uUTI [24]. IDSA 2025 guidelines outline treatment and management recommendations for cUTI only and therefore are not summarized here [9].

There is also growing interest in the use of non-antibiotic therapies for the management of recurrent uUTI and cystitis. Antibiotic-sparing strategies are crucial in preserving the use of existing antimicrobial agents during a time of increasing antimicrobial resistance and deceleration in new antimicrobial drug developments [36]. Behavioral and personal hygiene measures constitute routine self-care measures advice provided to patients to reduce the risk of recurrent UTI, including the following: adequate hydration; avoidance of douching, tampon use, and cotton underwear; wiping vulval and perineal areas from front to back after defecation; and avoiding delay of habitual and post-coital urination [36]. Non-antibiotic therapies that have shown strong evidence in preventing recurrent UTI include vaginal estrogens for perimenopausal, menopausal, and postmenopausal females, and methenamine hippurate; these strategies are beginning to be incorporated into national guidelines [8, 36].

Other antibiotic-sparing strategies which require further investigation in larger, well-powered, randomized controlled studies to confirm efficacy in the prevention of recurrent UTI include: strategies to prevent bacterial adherence in the bladder, eg cranberry, D-mannose, intravesical hyaluronic acid with or without chondroitin sulfate, pentosan polysulfate, and phytotherapeutics; strategies to modify urinary or vaginal pH and microbiota, eg ascorbic acid or probiotics; vaccines that use heat-inactivated whole cells, lyophilized lysates, or virulence factors of the uropathogenic organism; competitive inoculation, which involves intravesical instillation of less pathogenic uropathogens into the bladder to prevent infection with more virulent strains; and phage therapy, which disrupts bacterial biofilms [8, 36, 37].

## Impact of Urinary Tract Infections on Patients’ Quality of Life

UTIs can have a significant impact on patients’ quality of life and their healthcare experience, affecting productivity, finances, and activities of daily living. A nationwide healthcare practitioner (HCP) survey in the USA determined that 73% of participants missed days at work due to UTIs, 72% felt that UTIs affect their romantic/sexual relationships, 71% were concerned about experiencing another UTI, 66% experienced a financial burden, and 65% missed social events due to a UTI [38]. According to a survey of adult women with recurrent UTI, participants had a mean of 3.09 days sick leave per year due to UTIs, and felt limited in their daily activities for a mean of 3.45 days per year; missed work days can impact productivity and contribute to the indirect costs associated with recurrent UTI [1].

Interactions with HCPs and conversations surrounding antimicrobial prescribing can impact patients’ satisfaction with their care, including the patient being given enough time, feeling they are listened to, having tests and treatments explained to them, being involved in care decisions, and being treated with care and concern [39]. A survey in the UK demonstrated that healthcare practices prescribing 25% fewer antibiotics than the national mean had lower patient satisfaction, indicated by reductions of 0.5%–1.0% in patient satisfaction scores [39].

## CONCLUSION

The uUTI patient journey is complex due to a multitude of factors impacting treatment decisions and the patient experience. It is important for physicians to aim to treat the acute event using appropriate targeted therapy, knowing the local epidemiology, trying to reduce recurrence, reducing collateral damage to the gut microbiome, and improving overall patient satisfaction. Understanding and identifying risk factors for treatment failure is an important factor in treatment selection; these include risk factors for uUTI recurrence and AMR. The global increase in *E. coli* resistance, particularly due to ESBLs, may limit treatment options for many patients with uUTI; however, with the recent approval of new antibiotics, physicians have more options and can identify the most suitable treatment for each individual patient.
